# Does preoperative positron emission tomography with computed tomography predict nodal status in endometrial cancer? A pilot study

**DOI:** 10.3747/co.v15i3.176

**Published:** 2008-06

**Authors:** D. Nayot, J.S. Kwon, M.S. Carey, A. Driedger

**Affiliations:** * Department of Obstetrics and Gynecology, University of Toronto, ON; † Department of Gynecologic Oncology, M.D. Anderson Cancer Center, Houston, TX, U.S.A; ‡ Department of Systems Biology, M.D. Anderson Cancer Center, Houston, TX, U.S.A; § Department of Nuclear Medicine, University of Western Ontario, ON

**Keywords:** Positron emission tomography, pet, computed tomography, ct, endometrial cancer, preoperative, lymph nodes

## Abstract

Fewer than 20% of women with endometrial cancer have positive nodes, and an accurate noninvasive imaging modality to assess lymph node status would be helpful in selecting those who need lymphadenectomy. The objective of this pilot study was to evaluate positron emission tomography with computed tomography (pet–ct) in predicting nodal status before surgery for endometrial cancer. Twelve patients were enrolled at a single tertiary care centre. The sensitivity and specificity of preoperative pet–ct in predicting nodal status were 53.3% and 99.6% respectively. Using pet–ct, all metastatic nodes may not necessarily be detected, especially nodes with microscopic disease. The sensitivity of this imaging modality has to be improved before it can routinely be used in the preoperative evaluation of endometrial cancer.

## 1. INTRODUCTION

Endometrial cancer is the most common gynecologic malignancy and the fourth most common cancer affecting women, with approximately 4100 new cases estimated in Canada in 2007^1^. Surgical staging, which includes hysterectomy and bilateral salpingo-oophorectomy (hbso), with pelvic and para-aortic lymphadenectomy, is recommended for patients at high risk for extrauterine disease. However, that procedure requires referral to a gynecologic oncologist at a tertiary care center and is associated with potential complications, including nerve and vessel injury and lymphocyst development 2. Fewer than 20% of endometrial cancer cases are estimated to have positive nodes^3^. An accurate, noninvasive, preoperative imaging modality to assess lymph node status would therefore be very helpful in selecting patients for a surgical staging procedure that would include lymphadenectomy.

The novel imaging modality^18^F-fdg (fluorodeoxyglucose) positron emission tomography (pet) identifies tumours by differentiating the increased glycolytic rate of neoplastic cells as compared with normal cells. The concurrent use of computed tomography (ct) provides precise localization of the tumour. Data on preoperative combined pet–ct in predicting lymph node status in endometrial cancer are limited. We conducted a prospective pilot study of ^18^F-fdg pet with ct in assessing pelvic and para-aortic lymph nodes in women with high-risk endometrial cancer before surgical staging.

## 2. PATIENTS AND MATERIALS

This prospective cohort study was approved by the Health Sciences Research Ethics Board of the University of Western Ontario, Canada. The use of ^18^F-fdg for the study was approved by Health Canada.

All eligible patients received a pet–ct scan within 4 weeks before their surgery. The protocol included an intravenous dose of 555 MBq ^18^F-fdg, and at 60 minutes post injection of the fdg tracer, whole-body pet with ct images of the abdomen and pelvis. At 90 minutes from injection, furosemide 40 mg was given to facilitate excretion of the ^18^F-fdg from the urinary tract. At 120–150 minutes, patients underwent delayed imaging of the abdomen and pelvis. All pet–ct scans were interpreted by a single nuclear medicine consultant physician (A.D.). The examinations were performed on a Discovery DST pet–ct scanner (GE Medical Systems, Milwaukee, WI, U.S.A.). Lymph nodes were classified as “positive” or “negative” according to increased fdg uptake in the pelvic or para-aortic nodal regions, and if positive, a corresponding anatomic location was assigned according to the ct images. Standardized uptake values (suv) were obtained, but no specific suv threshold for lymph node positivity was found.

All surgical staging procedures were performed by one of two gynecologic oncologists (J.S.K., M.S.C.). The surgeons were not blinded to the preoperative pet–ct; however, all surgeries were completed systematically, beginning with hbso and followed by complete pelvic and para-aortic lymphadenectomy from the deep circumflex iliac vein inferiorly to the renal vessels superiorly. The pathology report included lymph node counts and indication of the presence or absence of metastatic disease. Test characteristics of sensitivity, specificity, and positive (ppv) and negative (npv) predictive values at both the patient and the nodal level were calculated.

## 3. RESULTS

Between May 2004 and November 2006, 12 patients were enrolled. Mean age was 62.6 years (range: 55–74 years). From these 12 patients, a total of 244 lymph nodes were removed, including 167 pelvic nodes (15 per patient on average) and 77 para-aortic nodes (7 per patient on average). Nodes were positive in 3 patients (stage iiic), and negative in 9 (stage i or ii disease).

At the patient level, preoperative pet–ct accurately predicted nodal status (positive or negative) for all patients in the study. All 3 who had metastatic nodal disease (stage iiic) were correctly identified by increased fdg uptake in the pelvic or para-aortic nodal regions on preoperative pet–ct, and all 9 patients who did not have metastatic nodal disease (that is, stage i or ii) were correctly identified as having disease confined to the uterus. As a result, the sensitivity, specificity, and ppv and npv for pet–ct in predicting nodal status at the patient level were all 100%.

However, at the nodal level, we observed discrepancies between pet–ct and surgical and pathologic results in 2 patients. In one patient of these patients, increased fdg uptake was seen in 1 left pelvic node (suv 16.1) and 4 left para-aortic nodes (average suv 16.0). At surgery, the left pelvic and para-aortic nodes were grossly abnormal (enlarged). Final pathology confirmed metastatic disease in 2 left pelvic nodes, 3 left para-aortic nodes, and 1 right pelvic node, the latter of which was normal in size. In the other patient, increased fdg uptake was seen in 1 left pelvic node (suv 5.5). At surgery, a cluster of 5 enlarged left pelvic nodes was found. Final pathology confirmed metastatic disease in 5 left pelvic nodes and 1 right pelvic node. Only one other patient had a positive pet–ct, with increased fdg uptake in 3 para-aortic nodes (average suv 7.2). Those nodes were grossly normal at surgery, but were confirmed as having metastatic disease on final pathology.

There was no consistent relationship between suv and the extent or size of metastatic nodal disease. At the nodal level, pet–ct showed a sensitivity of 53.3% (8/15) and a specificity of 99.6% (222/229) for detecting pelvic and para-aortic nodes. The ppv was 88.9% (8/9), and the npv was 97.0% (235/244).

## 4. DISCUSSION

The results of the present study suggest that the sensitivity of pet–ct may be insufficient for routine preoperative use of this imaging modality. Very few studies have considered pet evaluation of lymph nodes in endometrial cancer specifically. Horowitz *et al.* reported sensitivity and specificity of 67% and 94% respectively 4, but only 2 patients had metastatic nodes, and 1 had a false-negative pet study with a 1.0-cm metastatic pelvic node. Chao *et al.* reported sensitivity of 73% and specificity of 95% for metastatic pelvic nodes with pet 5; however, when pet was interpreted with concurrent magnetic resonance imaging (mri) or ct (that is, pet–ct, as in the present study), sensitivity and specificity improved to 85% and 100% respectively. On the other hand, Suzuki *et al.* reported a sensitivity of 0% for pet in detecting nodal metastases in endometrial cancer 6. All of the patients in that study had metastatic foci smaller than 6 mm, but ct or mri correctly identified 2 of 5 patients with metastatic pelvic nodes and the 1 patient with metastatic para-aortic nodal disease.

Using pet–ct, a single enlarged metastatic node may not be able to be distinguished from a cluster of meta-static nodes—a problem that may not be clinically significant, because preoperative identification of even 1 metastatic node would prompt complete lymphadenectomy. However, 2 patients in our study had a single right pelvic node with microscopic tumour that did not have increased fdg uptake on pet–ct. In both cases, the right pelvic nodes were grossly normal at surgery. If these had been the only metastatic nodes present, the pet–ct would have been falsely negative, and the nodes would have been missed if these patients did not have a complete lymphadenectomy.

Ideally, pet–ct should predict lymph node status accurately enough to influence decision-making about surgical staging. It must be sufficiently sensitive that it can reliably select patients for lymphadenectomy, but it must also have a negative predictive value sufficiently high that it can obviate the need for lymphadenectomy.

## 5. CONCLUSIONS

In this small pilot study, preoperative pet–ct appeared insufficiently sensitive to detect all metastatic nodes, especially those with microscopic foci of disease. At this time, the evidence that pet–ct can reliably predict nodal metastases before surgery is insufficient, and refinement of this imaging modality would be required before it can routinely be used in the preoperative evaluation of endometrial cancer.

## References

[1] Canadian Cancer Society and the National Cancer Institute of Canada (2005). Canadian Cancer Statistics 2005.

[2] Mohan DS, Samuels MA, Selim MA (1998). Long-term outcomes of therapeutic pelvic lymphadenectomy for stage i endometrial adenocarcinoma. Gynecol Oncol.

[3] Lo KW, Cheung TH, Yu MY, Yim SF, Chung TK (2003). The value of pelvic and para-aortic lymphadenectomy in endometrial cancer to avoid unnecessary radiotherapy. Int J Gynecol Cancer.

[4] Horowitz NS, Dehdashti F, Herzog TJ (2004). Prospective evaluation of fdg-pet for detecting pelvic and para-aortic lymph node metastasis in uterine corpus cancer. Gynecol Oncol.

[5] Chao A, Chang TC, Ng KK (2006). ^18^F-fdg pet in the management of endometrial cancer. Eur J Nucl Med Mol Imaging.

[6] Suzuki R, Miyagi E, Takahashi N (2007). Validity of positron emission tomography using fluoro-2-deoxyglucose for the preoperative evaluation of endometrial cancer. Int J Gynecol Cancer.

